# Redirecting immunity *via* covalently incorporated immunogenic sialic acid on the tumor cell surface†
†Electronic supplementary information (ESI) available: The synthesis of DNP conjugated sialic acids, western blot analysis, treatment and analysis of sugar-treated cells and mice. See DOI: 10.1039/c5sc04133c


**DOI:** 10.1039/c5sc04133c

**Published:** 2016-02-24

**Authors:** Bijuan Lin, Xuanjun Wu, Hu Zhao, Yunpeng Tian, Jiahuai Han, Jian Liu, Shoufa Han

**Affiliations:** a State Key Laboratory for Physical Chemistry of Solid Surfaces , Department of Chemical Biology , College of Chemistry and Chemical Engineering , The Key Laboratory for Chemical Biology of Fujian Province , The MOE Key Laboratory of Spectrochemical Analysis & Instrumentation, and Innovation Center for Cell Signaling Network , Xiamen University , Xiamen , 361005 , China . Email: shoufa@xmu.edu.cn ; Tel: +86-0592-2181728; b State Key Laboratory of Cellular Stress Biology , Innovation Center for Cell Signaling Network , School of Life Sciences , Xiamen University , Xiamen , 361005 , China

## Abstract

Techniques eliciting anti-tumor immunity are of interest for immunotherapy. We herein report the covalent incorporation of a non-self immunogen into the tumor glycocalyx by metabolic oligosaccharide engineering with 2,4-dinitrophenylated sialic acid (^DNP^Sia). This enables marked suppression of pulmonary metastasis and subcutaneous tumor growth of B16F10 melanoma cells in mice preimmunized to produce anti-DNP antibodies. Located on the exterior glycocalyx, ^DNP^Sia is well-positioned to recruit antibodies. Given the high levels of natural anti-DNP antibodies in humans and ubiquitous sialylation across many cancers, ^DNP^Sia offers a simplified route to redirect immunity against diverse tumors without recourse to preimmunization.

## Abstract 2

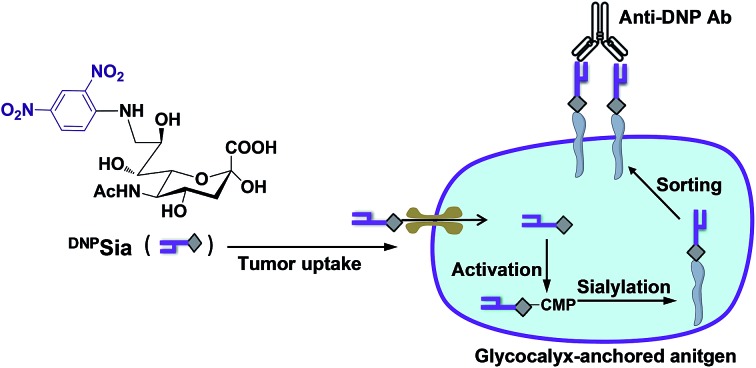
Anti-tumor immunity was achieved *via* metabolically incorporated non-self antigen-labelled sialic acid on the tumor surface glycocalyx.

## Introduction

Immune systems eradicate deleterious “non-self” cells while saving “self” cells by sensing cell surface biomarkers. As cancers evade the immune system surveillance, extensive effort has been devoted to redirect immunity against tumors by targeting biomarkers that are often nonimmunogenic. For instance, cytotoxic T cells genetically engineered with chimeric antigen receptors have been actively explored for targeting tumor surface antigens.^1^ However, challenges remain with these self-antigen targeted therapies owing to the presence of self-antigens on normal tissues and the inability to turn off persistent T cell activity.^2^ Alternatively, ligand–antigen adaptor molecules that bind avidly to tumor surface receptors (*e.g.* the folate receptor) enable exogenous antigens displayed on tumors to trigger immune responses.^3^ Albeit powerful, these approaches rely on tumor-specific high affinity receptors that are often not defined in many cancers.

**Scheme 1:**
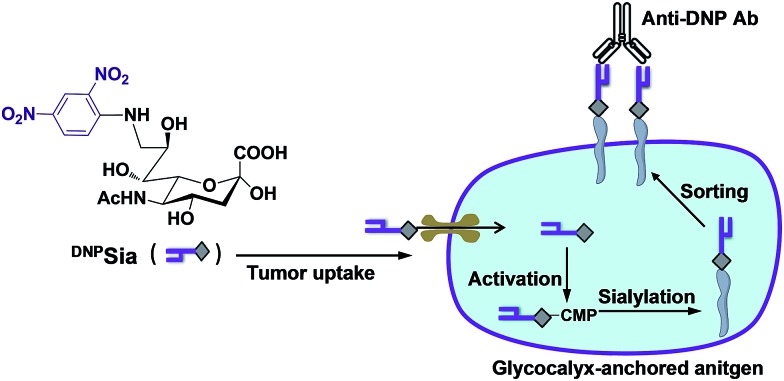
Schematic for the incorporation of the non-self antigen into glycocalyx. ^DNP^Sia taken up by the tumors is metabolically transferred to glycoconjugates. The neosialoconjugates sorted to the cell surface enable ^DNP^Sia to be well-positioned to trigger immunity.

Mammalian cells are covered with a dense layer of glycans, known as glycocalyx, which mediates diverse cellular events, such as immunological recognition and cancer metastasis.^4^ Sialic acids (Sia) are a family of 9-carbon monosaccharides commonly located at the cell surface glycan termini.^5^ Hypersialylation contributes to the metastatic potentials of many cancers,^6^ and facilitates tumor evasion of the immune system surveillance.^7^ Cell surface sialosides have been engineered with exogenous *N*-acyl mannosamines, metabolic precursors of Sia.^8^ However, the oligosaccharide engineering approach using peracetylated sugars is of low cell type- or tissue-specificity, leading to expression of *N*-acyl Sia in diverse tissues in animals.^9^ We recently observed a marked propensity of tumors to take up Sia derivatives with selected substitutions at C-9 in mice.^10^ We herein report covalent incorporation of a non-self immunogen into tumor glycocalyx with DNP-conjugated Sia (^DNP^Sia) to redirect immunity against tumors. ^DNP^Sia effectively accumulates in tumors and is covalently installed into the cell surface glycocalyx by an endogenous sialylation pathway. Mice preimmunized with DNP-labelled keyhole limpet hemocyanin (^DNP^KLH) display marked antitumor effects against ^DNP^Sia-displaying B16F10 murine melanoma cells. Complementing receptor–ligand affinity based tumor coatings,^3^ this work suggests an alternative approach for tumor therapy *via* a metabolically incorporated non-self antigen (Scheme 1).

## Results and discussion

### Metabolic incorporation of ^DNP^Sia on the cell surface glycocalyx

Sia chemically modified with C-9 substitutions are often compatible with cellular sialylation, leading to the incorporation of abiotic Sia into glycoconjugates. For instance, FITC-labelled Sia sialylates proteins in permeabilized CHO cells.^11^ Sia bearing an aromatic azide can be effectively incorporated into CD22 on the B cell surface.^12^ We recently observed that Sia with hydrophobic groups at C-9 preferentially accumulates in tumors in mice.^10^ Encouraged by these observations, we explored the efficacy of ^DNP^Sia incorporated on glycocalyx for tumor suppression.

**Fig. 1:**
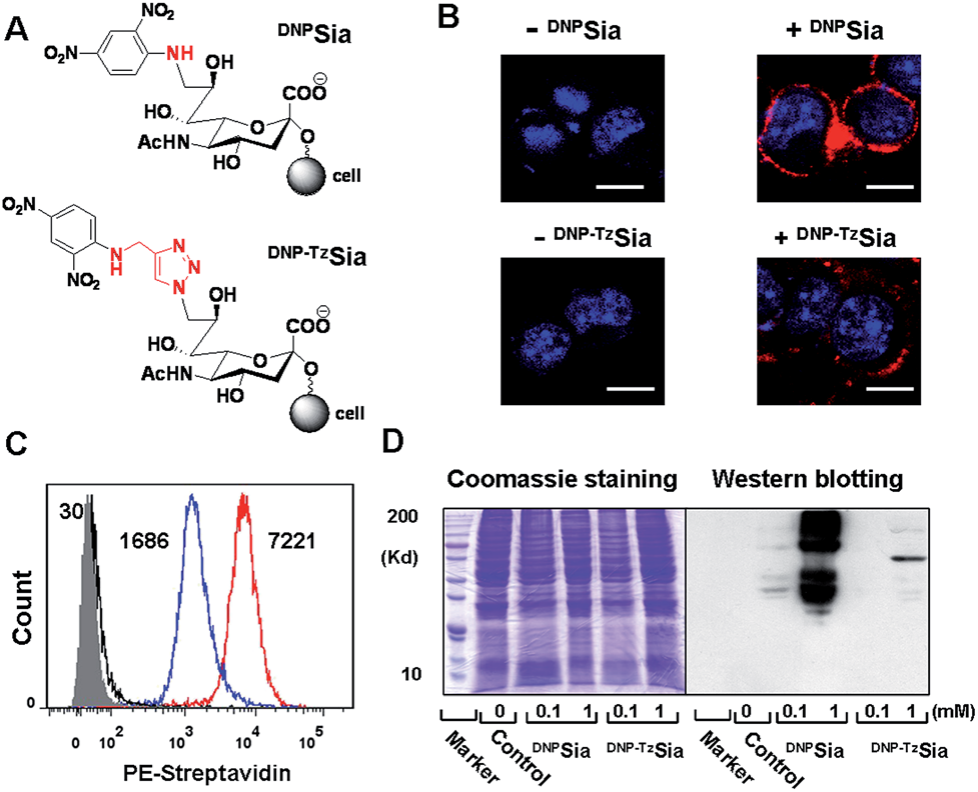
Incorporation of DNP-Sia diads into cell glycocalyx. Chemical structures of sialosides of ^DNP^Sia and ^DNP–Tz^Sia displayed on a cell surface (A). B16F10 cells treated with the methyl esters of ^DNP–Tz^Sia or ^DNP^Sia (0, 1 mM) were stained with biotin-labelled anti-DNP Ab, PE-labelled streptavidin, and DAPI specific for the nucleus, and then analysed by confocal fluorescence microscopy (B) or flow cytometry (C) with the mean channel fluorescence (MF) indicated. Bars: 10 μm. (D) Western blot of the lysate of cells treated with the DNP-Sia diads. Protein loading was verified by Coomassie Blue staining.

We first probed the impact of the spacers of DNP-Sia diads on the cell surface sialoside expression. ^DNP^Sia featuring an amino spacer was synthesized from the nucleophilic aromatic substitution of 2,4-dinitrofluorobenzene with 9-amino-Sia whereas ^DNP–Tz^Sia bearing a 1,2,3-triazole (Tz) linker was synthesized by the copper(i)-catalyzed azide–alkyne cycloaddition of 9-azido-Sia with 2,4-dinitro-1-propargylamino-benzene (Fig. 1A, Scheme S1 and S2, ESI†). B16F10 cells, poised to hypersialylation,^13^ were cultivated in Dulbecco's modified Eagle medium (DMEM) spiked with the methyl esters of ^DNP–Tz^Sia or ^DNP^Sia (Scheme S1 and S2, ESI†), and then stained with biotin-labelled anti-DNP antibodies (Ab) and phycoerythrin (PE)-labelled streptavidin to probe the degrees of cell surface DNP. Confocal microscopic images reveal bright PE fluorescence confined to the plasma membranes of the cells treated with the methyl esters of ^DNP^Sia or ^DNP–Tz^Sia, whereas no signal is observed in the control cells (Fig. 1B). Flow cytometry analysis revealed a 4-fold enhancement of cell surface ^DNP^Sia (MF = 7221) compared to ^DNP–Tz^Sia (MF = 1686) (Fig. 1C). Western blotting confirms the high abundance of ^DNP^Sia-bearing proteins over ^DNP–Tz^Sia-bearing proteins (Fig. 1D). These results validate the covalent incorporation of ^DNP^Sia into the glycocalyx with superior efficacy relative to ^DNP–Tz^Sia. ^DNP^Sia was also effectively installed on the cell surface of Raw 264.7 macrophages, HeLa cells, L929, SMMC-7721 and U87-MG cells (Fig. S1, ESI†), demonstrating the compatibility of ^DNP^Sia with sialylation pathways of diverse cancer cell lines. The immunostaining of cell surface DNP shows that glycocalyx-anchored ^DNP^Sia is well-positioned to recruit anti-DNP Ab. We then monitored the temporal changes of cell surface ^DNP^Sia on B16F10 cells cultivated in fresh DMEM. Albeit decaying over time, the levels of glycocalyx-anchored ^DNP^Sia remained high after 24 h incubation (Fig. S2, ESI†). In addition, cells surface ^DNP^Sia was shown to be more resistant to sialidase-mediated hydrolysis relative to Sia (Fig. S3, ESI†), which is beneficial for *in vivo* immunotherapy.

### Anti-tumor effects of glycocalyx-anchored ^DNP^Sia in mice

**Fig. 2:**
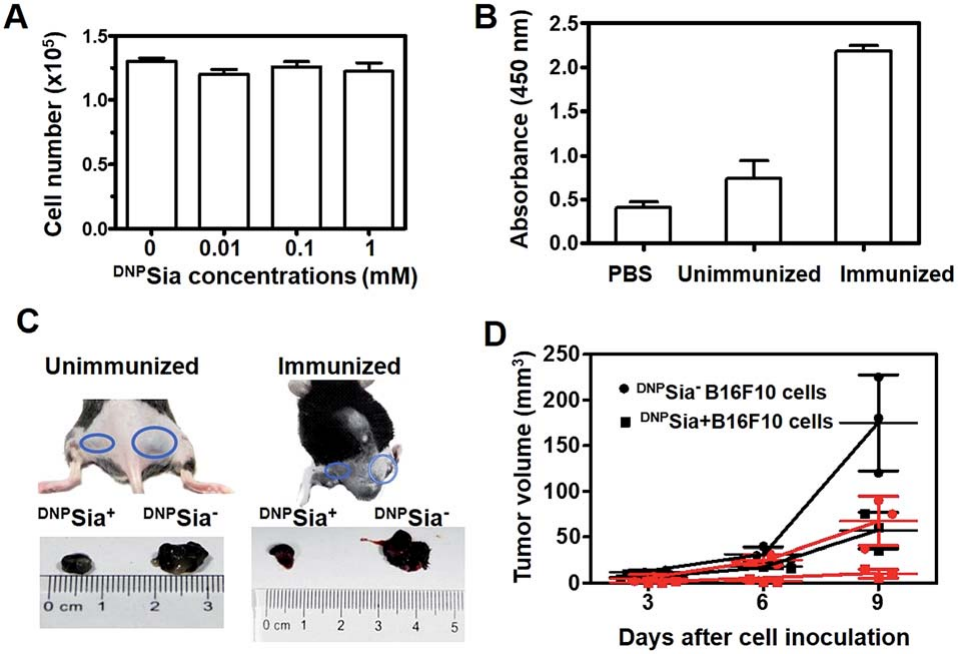
^DNP^Sia mediated anti-tumor responses in mice. (A) Effects of ^DNP^Sia on cell proliferation. B16F10 cells pretreated with ^DNP^Sia (0–1 mM) were cultured in fresh DMEM for 24 h prior to cell number determination. (B) ELISA of anti-DNP Ab in the serum from C57BL/6 mice untreated or treated with an injection of ^DNP^KLH. (C) Suppressed growth of inoculated ^DNP^Sia^+^ B16F10 cells over ^DNP^Sia-free cells that are injected in opposite flanks of mice. Tumors were excised 7 days post inoculation. (D) Anti-DNP Ab mediated inhibition of ^DNP^Sia^+^ B16F10 cells and ^DNP^Sia^–^ cells subcutaneously inoculated in mice unimmunized (in dark) or immunized with ^DNP^KLH (in red). Assays were performed in triplicate each using 3 mice. Error bars represent ±SD of experimental data on a representative assay.

Next, ^DNP^Sia was assessed for its influence on tumor cell proliferation *in vitro*. B16F10 cells pretreated without or with ^DNP^Sia were maintained in fresh DMEM for 24–72 h. No detrimental effects of ^DNP^Sia were observed on cell growth (Fig. 2A and S4, ESI†). With the negligible effects on cell growth *in vitro*, ^DNP^Sia was then explored for its capability to redirect immunity *in vivo*. C57BL/6 mice were immunized with ^DNP^KLH and subsequently boosted with another injection. An enzyme-linked immunosorbent assay (ELISA) shows that the level of anti-DNP Ab in the serum from treated mice is 5-fold higher than in the untreated mice (Fig. 2B), proving induction of DNP-specific Ab by injected ^DNP^KLH. ^DNP^Sia-displaying B16F10 cells (^DNP^Sia^+^) and cells devoid of ^DNP^Sia (^DNP^Sia^–^) were subcutaneously inoculated into the left or right flank of ^DNP^KLH-treated mice, respectively. The tumor from the ^DNP^Sia^+^ cells exhibits about 75% volume reduction compared to that from the ^DNP^Sia^–^ tumor 7 days after inoculation, whereas the tumor formation of the ^DNP^Sia^–^ cells with natural Sia was largely unaffected in DNP-immunized mice relative to unimmunized mice (Fig. 2C). In unimmunized mice, the tumor from ^DNP^Sia^+^ cells was about 50% smaller relative to that from the ^DNP^Sia^–^ cells, whereby retarded growth of the ^DNP^Sia^+^ tumor is likely due to endogenous low levels of anti-DNP Ab (Fig. 2B). In addition, time course monitoring reveals consistent and obvious suppression of tumor formation from implanted ^DNP^Sia^+^ cells over ^DNP^Sia^–^ cells in ^DNP^KLH-immunized mice (Fig. 2D) in the early stage, demonstrating synergistic effects of anti-DNP Ab and ^DNP^Sia on the anti-tumor response.

**Fig. 3:**
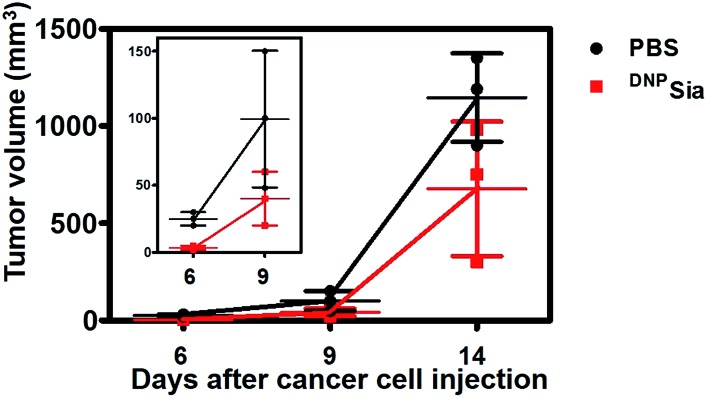
Tumor suppression with intravenously injected ^DNP^Sia in mice. ^DNP^KLH-immunized C57BL/6 mice were subcutaneously inoculated with B16F10 cells, and then treated with a tail-vein injection of PBS (100 μL) or ^DNP^Sia (30 mg kg^–1^) on the 3^rd^, 6^th^, and 9^th^ day post cell inoculation. The tumor volumes were monitored over time. The inset shows the correlation of early stage tumor volume *vs.* time. The assays were performed in triplicate, using 3 mice each time. The error bars represent ±SD of the experimental data on a representative assay.

To further evaluate the therapeutic scope of this approach, ^DNP^KLH-immunized mice subcutaneously inoculated with B16F10 cells were treated with a tail-vein injection of PBS or ^DNP^Sia after tumor transplantation. ^DNP^Sia treatment resulted in a 50–90% reduction in the tumor volume in the early stage (6–9 days after cell inoculation) compared to the control mice treated with PBS (Fig. 3), proving the effectiveness of ^DNP^Sia for systemic tumor suppression.

**Fig. 4:**
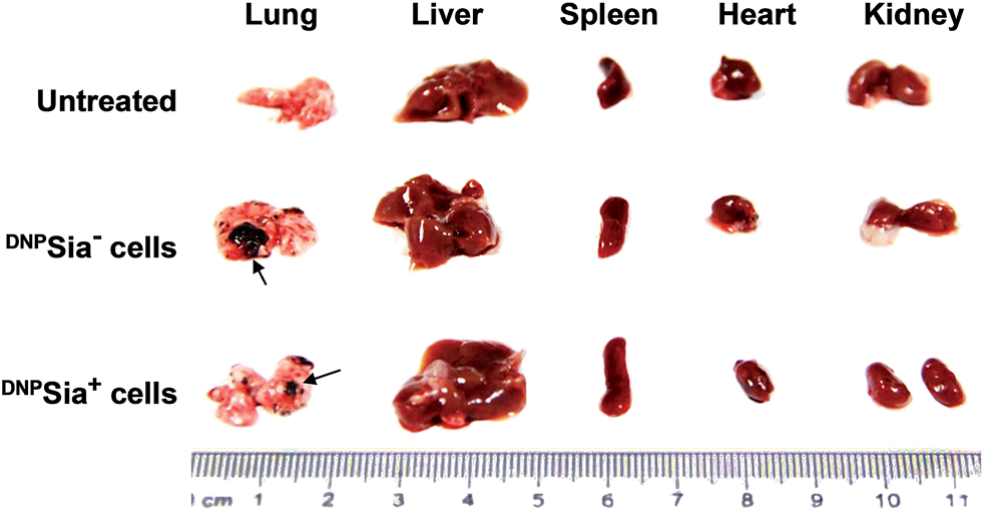
Glycocalyx-anchored ^DNP^Sia decreases pulmonary metastasis of B16F10 cells in mice. ^DNP^KLH-immunized C57BL/6 mice were untreated or treated with ^DNP^Sia^–^, or ^DNP^Sia^+^ B16F10 cells by tail-vein injection. The organs were excised 7 days after injection. The arrows denote metastases.

Metastasis is a major cause of cancer-associated mortality. Cell surface sialosides are critical for cancer metastasis, promoting the use of sialyltransferase inhibitors to decrease cancer sialylation.^14^ We therefore evaluated the effects of ^DNP^Sia on B16F10 metastasis with an experimental pulmonary metastasis model. ^DNP^Sia^+^ B16F10 cells were injected into ^DNP^KLH-immunized mice *via* the tail-vein. Lungs and representative organs were isolated 7 days after cell administration. The metastasis in the lung from mice treated with ^DNP^Sia^+^ cells is significantly smaller than that from mice treated with ^DNP^Sia^–^ cells (Fig. 4 and S5, ESI†), revealing the capability of ^DNP^Sia to inhibit metastasis. Our results suggest an alternative approach against metastasis with chemically modified Sia on the cell surface.

Compared with affinity based tumor decoration,3b,d,^15^ we employ antigens covalently installed on glycocalyx *via* a metabolic sialylation pathway to elicit antitumor responses. Historically, sialosides with selected *N*-acyl groups at C-5 have been reported by Guo's group and Bertozzi's group to be more immunogenic than natural sialoside.^16^ The feasibility of this approach has been examined in both cell and mice models with exogenous *N*-acyl mannosamines, metabolic precursors of C-5-*N*-acyl Sia.16e,^17^ This abiotic Sia-mediated cancer therapy entails tandem preimmunizations to elicit Ab specific to the abiotic sialosides, and subsequent tumor expression of the abiotic Sia. These studies lay the foundation for unnatural Sia mediated immunotherapy. One percent of circulating antibodies in the human body binds DNP.^18^ Of note, our approach directly uses DNP-bearing Sia for metabolic tumor engineering, and offers simplified immunotherapy by recruiting high levels of natural anti-DNP Ab in humans without recourse to preimmunization.

### Biodistribution of ^DNP^Sia in tumor-bearing mice

**Fig. 5:**
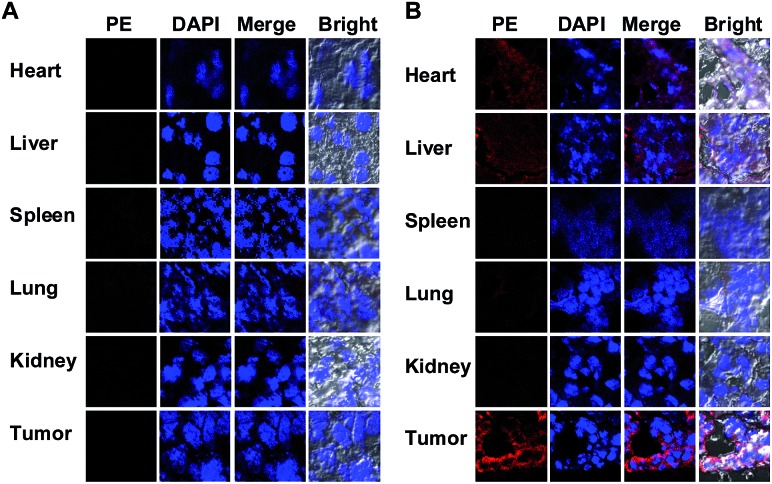
*In vivo* distribution of ^DNP^Sia. C57BL/6 mice bearing subcutaneous B16F10 tumors were injected *via* the tail-vein with PBS (A), or ^DNP^Sia (60 mg kg^–1^) (B). The tumors (10 mm^3^) and organs were excised 1 h post-injection, sectioned, and stained with biotin-labelled anti-DNP Ab, PE-labelled streptavidin and DAPI prior to fluorescence analysis.

**Fig. 6:**
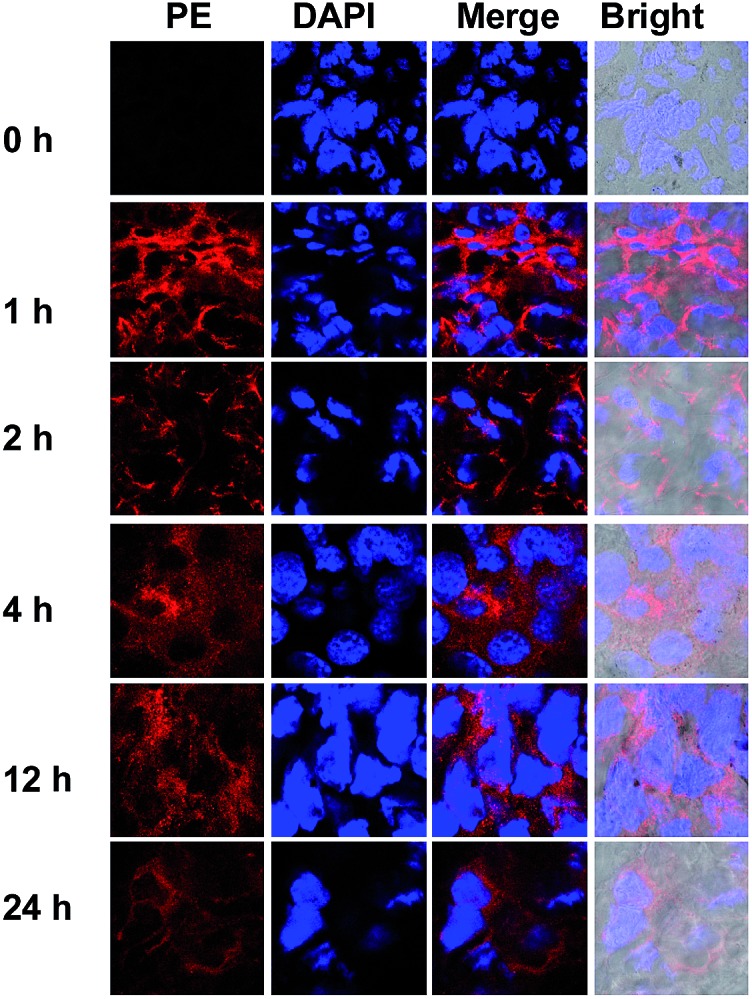
Temporal retention of ^DNP^Sia in tumors. C57BL/6 mice with B16F10 tumors (10 mm^3^) were injected with ^DNP^Sia (60 mg kg^–1^) in the tail-vein. At 0–24 h after the injection, the tumors were excised, sectioned, and stained with biotin-labelled anti-DNP Ab, PE-labelled streptavidin, and DAPI prior to fluorescence detection.

To probe the ^DNP^Sia biodistribution, C57BL/6 mice bearing subcutaneous B16F10 tumors were treated with ^DNP^Sia by tail-vein injection. The tumors and selected organs were excised 1 h post-injection and examined for ^DNP^Sia expression. Immunostaining reveals intense fluorescence in the tumors whereas negligible to moderate fluorescence is present in the kidney, heart, spleen, lung and liver (Fig. 5), showing that ^DNP^Sia is preferentially taken up and incorporated into glycoconjugates by tumors in mice. ^DNP^Sia displayed compromised tumor accumulation as the tumor volume increases (data not shown). A time course study shows that the tumor-associated ^DNP^Sia decreased over time and yet remained substantial 24 h post-injection (Fig. 6). By contrast, ^DNP^Sia incorporated in the heart and liver decreased to baseline levels 4 h after sugar administration (Fig. S6, ESI†), suggesting *in vivo* clearance of non-self antigens, which offers a means to temporarily turn off immunity and thus is beneficial for decreased systemic toxicity after treatment. Although B16F10 is a cell line featuring oversialylation,^13^ the elevated expression of the tumor surface ^DNP^Sia sialoside might also benefit from rapid cell division and glycoprotein biosynthesis in tumor cells relative to normal tissue and organs. The lower levels of ^DNP^Sia in heart tissue could trigger off-target immune responses. In the future, the selective expression of ^DNP^Sia on tumors could be potentiated with the aid of emerging vectors for the tumor-specific delivery of Sia.14b,^19^

### Cytotoxicity of ^DNP^Sia

Low toxicity is critical for agents to be administered *in vivo*. To probe the systemic toxicity, ^DNP^Sia was injected into healthy mice by the tail-vein at a dose of 300 mg kg^–1^, which is 10-fold higher than the dose used for the systemic tumor suppression mentioned in Fig. 3. No signs of abnormal behavior or death were observed in the mice up to 14 days after injection. Histological analysis reveals that the morphologies of the organs from mice untreated or treated with ^DNP^Sia were virtually identical (Fig. S7, ESI†), indicating that ^DNP^Sia is of low systemic toxicity.

## Conclusions

We have demonstrated the use of ^DNP^Sia, a non-self immunogen tagged monosaccharide, to trigger immunity against tumors in mice. ^DNP^Sia is preferentially taken up by tumors and then metabolically incorporated into the cell surface, enabling marked suppression of pulmonary metastasis of ^DNP^Sia-bearing B16F10 melanoma cells and suppression of subcutaneous tumor formation by intravenously injected ^DNP^Sia in ^DNP^KLH-immunized mice. Given the high levels of natural anti-DNP antibodies in humans,18*a*^DNP^Sia on the outmost glycocalyx is well-positioned to recruit pre-existing antibodies and might offer a simplified immunotherapy in humans without recourse to preimmunization. Compared to ligand–receptor affinity mediated tumor targeting,^3^ our approach takes advantage of the widespread cellular sialylation pathway to covalently install non-self antigen conjugated Sia on the tumor glycocalyx, which, in principle, could increase the immunogenicity of diverse tumors with a broad range of immunogens.

## Supplementary Material

Supplementary information
