# Supplementary material for: Redirecting immunity via covalently incorporated immunogenic sialic acid on the tumor cell surface
Source: Chem Sci. 2016 Feb 24;7(6):3737–41. doi: 10.1039/c5sc04133c (PMC6008587; doi:10.1039/c5sc04133c)
Supplement: Supplementary file 1 [file SC-007-C5SC04133C-s001.pdf]

Electronic Supplementary Material (ESI) for Chemical Science.
This journal is © The Royal Society of Chemistry 2016

Supporting Information

Redirecting immunity via covalently incorporated immunogenic sialic acid on tumor cell surface

Xuanjun Wu,[a] Bijuan Lin,[a] Hu Zhao,[a] Yunpeng Tian,[a] Jiahuai Han,[b] Jian Liu,[a] and Shoufa Han[a,*]

[a]State Key Laboratory for Physical Chemistry of Solid Surfaces, Department of Chemical Biology, College of Chemistry and Chemical Engineering, the Key Laboratory for Chemical Biology of Fujian Province, The MOE Key Laboratory of Spectrochemical Analysis & Instrumentation, and Innovation Center for Cell Signaling Network, Xiamen University; [b]State key Laboratory of Cellular Stress Biology, Innovation Center for Cell Signaling Network, School of Life Sciences, Xiamen University, Xiamen, 361005, China; Tel: 86-0592-2181728; E-mail: shoufa@xmu.edu.cn

Experimental Procedures

Materials and methods:

Biotin-labeled anti-DNP Ab, PE-labeled streptavidin and HRP-conjugated streptavidin were purchased from Vector labs. DNP-conjugated keyhole limpet hemocyanin (^{DNP}KLH) and DNP-conjugated bovine serum albumin (^{DNP}BSA) were purchased from Alpha Diagnostic International Inc. HRP-conjugated goat anti-mouse Ab, TiterMax®Gold Adjuvant, and 3-(4,5-dimethylthiazol-2-yl)-2,5-diphenyltetrazolium bromide (MTT) were from Sigma. 4',6-Diamidino-2-phenylindole (DAPI) was obtained from Beyotime. DMEM and GIBCO fetal bovine serum (FBS) were purchased from Sigma. FBS was heat-inactivated at 56 ^{o}C for 30 min before use. All other chemicals were used as received from Alfa Aesar.

U87-MG, HeLa, Raw 264.7, B16F10, SMMC-7721 and L929 cells were obtained from American Type Culture Collection and grown at 37°C under 5% CO_2 in DMEM. Confocal microscopic images were performed on Leica SP5 using the following filters: *λex*@405 nm and *λem*@430-480 nm for DAPI; *λex*@488 nm and *λem*@530-630 nm for PE. Fluorescence images were merged by Photoshop CS 6.0. Graph data were processed with Graphpad Prism5 software. C57BL/6 mice were purchased from Xiamen University Laboratory Animal Center. All animal experiments were performed in accordance with the guidelines of Xiamen University's Animal Care and Use Committee.

Synthesis of 5-acetamido-9-(2',4'-dinitrobenzylamino)-3,5,9-trideoxy-*D*-glycero-r-*D*-galacto-non-2-ulopyranosonic acid (^{DNP}Sia).

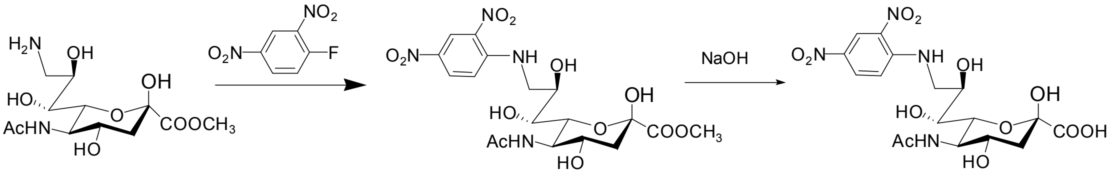

Scheme S1. Synthesis of ^{DNP}Sia.

5-Acetamido-9-amino-9-deoxy-3,5,9-trideoxy-D-glycero-r-D-galacto-2-nonulopyranosyl-onate

(9-amino-Sia methyl ester) (3.2 g) was added to a flask containing DMF (10 ml), 2,4-dinitro-1-fluorobenzene (2 g) and triethylamine (2 ml). The solution was maintained at rt for 30 min with stirring and then concentrated by rotary evaporation. The residue was purified by silica gel chromatography eluted with methylene chloride/methanol (10:1) to afford ^{DNP}Sia methyl ester (2.5 g) in 51% yield. ^{1}H-NMR (500 MHz, CD_3OD): δ 9.02 (1 H, d, *J* 2.6), 8.27 (1 H, dd, *J* 9.6, 2.6), 7.21 (1 H, d, *J* 9.6), 4.09 – 4.01 (2 H, m), 3.97 (1 H, td, *J* 8.1, 3.2), 3.88 (1 H, dd, *J* 13.6, 3.2), 3.81 (1 H, d, *J* 9.6), 3.77 (3 H, s), 3.52 (2 H, m, *J* 12.4, 8.4), 2.24 (1 H, dd, *J* 12.9, 4.9), 2.01 (3 H, s), 1.95 – 1.88 (1 H, m); ^{13}C-NMR (126 MHz, DMSO-d6): δ 175.22, 171.67, 150.06, 136.91, 131.55, 131.01, 124.70 , 116.21 , 96.68 , 72.04 , 71.99 , 69.58 , 67.69 , 54.34 , 53.22 , 47.70 , 40.72 , 22.61; HRMS ($C_{18}H_{24}N_4O_{12}$): calculated ($M+Na^+$): 511.1283, found: 511.1282.

To the solution of ^{DNP}Sia methyl ester (511 mg) in methanol (5 ml) was added 5 ml of aqueous NaOH (2 M). The solution was stirred at rt for 20 min and then neutralized with aqueous HCl (2 M) to neutral pH. The solution was concentrated and then purified by silica gel chromatography eluted with methylene chloride/methanol (2:1) to afford ^{DNP}Sia (250 mg) in 52% yield. ^{1}H NMR (500 MHz, CD_3OD): δ 9.03 (d, 1H, *J* = 2.6), 8.30 (dd, 1H, *J1* = 9.6, *J2* = 2.6), 7.24 (d, 1H, *J* = 9.6), 4.04 (d, 2H, *J* = 10.5), 3.97 (dd, 2H, *J1* = 20.3, *J2* = 6.7), 3.87 (dd, 1H, *J1* = 13.6, *J2* = 3.2), 3.57-3.52 (m, 1H), 3.50 (d, 1H, *J* = 8.9), 2.16 (dd, 1H, *J1* = 12.8, *J2* = 4.7), 2.03 (s, 3H), 1.90 (t, 1H, *J* = 12.1); ^{13}C-NMR (126 MHz, CD_3OD): δ 176.1, 173.3, 148.7, 135.5, 130.1, 129.7, 123.3, 115.0, 96.4, 70.6, 70.5, 68.3, 67.1, 52.6, 46.4, 40.4, 21.5; MS ($C_{17}H_{22}N_4O_{12}$): calculated ($M-H^+$): 473.1156, found: 473.1152.

Synthesis of *N*1-[5-Acetamido-4-carboxamido)- 3,5,9-trideoxy- D-glycero-r-D-galacto-non-2-ulopyranosyl-2-onic acid] methyl ester-4-[2-(4-hydroxy-3-nitrophenyl) acetamidomethyl]-[1,2,3] -triazole

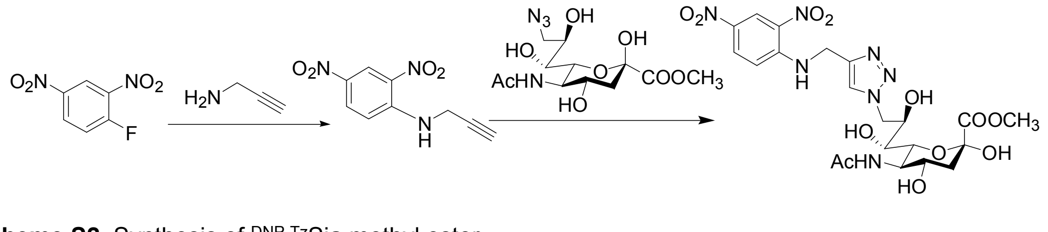

Scheme S2. Synthesis of $^{DNP\text{-}Tz}$Sia methyl ester.

Proparylamine (1 ml) and 2,4-dinitro-1-fluorobenzene (1.86 g) were added to acetonitrile (15 ml) containing N'N-diisopropylethylamine (1 ml). The solution was stirred for 2 h at 60 ℃and then cooled to give 2,4-dinitro-1- propargylaminobenzene as a high-purity yellow precipitation (1.8 g, 82%). ^{1}H-NMR (500 MHz, DMSO-d6): δ 8.86 (d, 1H, J = 2.7), 8.37 (dd, 1 H, J1 = 9.5, J2 = 2.7), 7.25 (d, 1H, J = 9.6), 4.35 (dd, 2H, J1 = 5.8, J2 = 2.4), 3.30 (t, 1H, J = 2.4). ^{13}C-NMR (126 MHz, DMSO-d6): δ 147.74, 136.01, 130.89, 130.42, 123.82, 116.12, 79.78, 75.23, 32.82.

To the solution of 9-azido-Sia methyl ester (1 g) and 2,4-dinitro-1-propargylaminobenzene (1 g) in tert-butanol (10 mL) were added sodium ascorbate (50 mg) and copper sulfate (10 mg) in water (5 ml). The reaction mixture was refluxed at 80 ℃ for 24 h. The solvent was then lyophilized and the residue solid was purified by silica gel chromatography using methylene chloride/methanol (10:1) as the eluent to give $^{DNP\text{-}Tz}$Sia methyl ester as a yellow solid (1 g, 59%). ^{1}H-NMR (500 MHz, CD_3OD): δ 9.02 (d, 1H, *J* = 2.1), 8.28 (dd, 1H, *J1* = 2.3, *J2* = 2.3), 8.04 (s, 1H), 7.29 (d, 1H, *J* = 9.6), 4.82 (s, 2H), 4.40 (q, 1H, *J* = 14.2), 4.09-4.01 (m, 3H), 3.83 (q, 1H, *J* = 15.1), 3.78 (s, 3H), 2.24 (dd, 1H, *J1* = 4.8, *J2* = 4.7), 2.01 (s, 3H), 1.89 (t, 1H, *J* = 12.5); ^{13}C-NMR (126 MHz, CD_3OD): 173.7, 170.5, 147.9, 143.1, 136.0, 130.6, 129.7, 125.6, 124.4, 114.8, 95.3, 70.6, 70.2, 69.3, 66.2, 53.7, 52.8, 52.0, 39.4, 38.1, 21.3; HRMS ($C_{21}H_{27}N_7O_{12}$): calculated (M+Na$^+$): 592.1610, found: 592.1617.

Incorporation of ^{DNP}Sia and $^{DNP\text{-}Tz}$Sia into cells surface and immunostaining of cell surface DNP

Time dependent incorporation of ^{DNP}Sia into differnet cell lines: U-87 MG, HeLa, Raw 264.7, SMMC-7721, L929 and B16F10 cells were cultured for 0-24 h in DMEM containing ^{DNP}Sia methyl ester (100 μM). The cells were harvested, washed with PBS and then incubated with biotin-labeled anti-DNP Ab (10 μg ml^{-1}) in PBS for 15 min at 4 °C. The cells were washed with PBS, and stained with PE-labeled streptavidin (10 μg ml^{-1}) for 15 min at 4 °C. The cells were washed with PBS and

then analyzed by confocal fluorescence microscopy.

Incorporation of $^{DNP\text{-}Tz}$Sia into different tumor cell lines: U-87 MG, HeLa, Raw 264.7, and B16F10 cells were cultured for 24 h in DMEM containing $^{DNP\text{-}Tz}$Sia methyl ester (100 μM). The cells were harvested, washed with PBS and then incubated with biotin-conjugated anti-DNP Ab (10 μg ml^{-1}) in PBS for 15 min at 4 °C. The cells were washed with PBS, and stained with PE-labeled streptavidin (10 μg ml^{-1}) for 15 min at 4 °C. The cells were washed with PBS and then analyzed by confocal fluorescence microscopy.

Optimized incorporation of ^{DNP}Sia and $^{DNP\text{-}Tz}$Sia into cells surface and staining of cell surface DNP: B16F10 cells that were cultivated with the ^{DNP}Sia methyl ester (100 μM), $^{DNP\text{-}Tz}$Sia methyl ester (100 μM), or no addition for 24 h were incubated with biotin-labeled anti-DNP Ab (10 μg ml^{-1}) in PBS for 15 min at 4 °C. The cells were washed with PBS, and stained with PE-labeled streptavidin (10 μg ml^{-1}) for 15 min at 4 °C. The cells were washed with PBS and then analyzed by confocal fluorescence microscopy and flow cytometry.

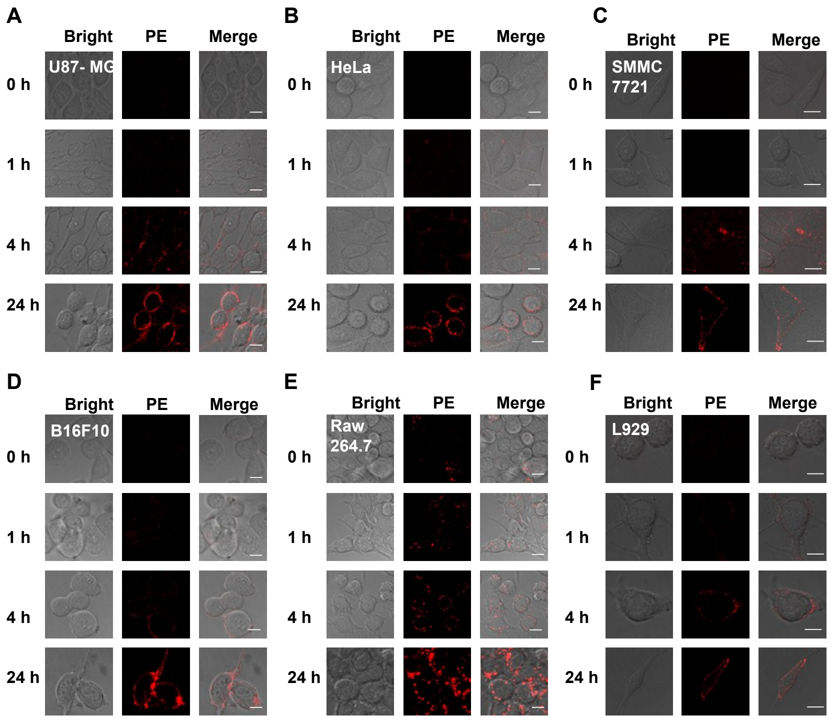

Fig. S1 Time course of metabolic incorporation of ^{DNP}Sia into cells surface glycoconjugates. U-87 MG, HeLa, SMMC-7721, B16F10, Raw 264.7 cells, and L929 cells were respectively cultivated with ^{DNP}Sia methyl ester (100 μM) in DMEM supplemented with 10% FBS for 0-24 h. The cells were then sequentially stained with biotin-labeled anti-DNP Ab (10 μg ml^{-1}) and PE-labeled streptavidin (10 μg ml^{-1}) for 15 min at 4 °C. The cells were washed with PBS and analyzed by a confocal fluorescence

microscope. Bar: 10 μm.

Retention of cells surface ^{DNP}Sia

Catabolism of cell surface ^{DNP}Sia: B16F10 cells were first cultured for 24 h in DMEM containing ^{DNP}Sia methyl ester (100 μM). The cells were harvested, washed with PBS and then cultivated in fresh DMEM for 0-24 h. At 0, 4, 24 h of incubation, the cells were aliquoted, washed with PBS and then stained with biotin-labeled anti-DNP Ab (10 μg ml^{-1}) in PBS for 15 min at 4 °C. The cells were washed with PBS, and stained with PE-labeled streptavidin (10 μg ml^{-1}) for 15 min at 4 °C. The cells were washed with PBS and then analyzed by flow cytometry. Enymatic hydrolysis of cell surface DNPSia: B16F10 cells were culrured with methyl esters of ^{DNP}Sia or Sia (1 mM) for 24 h in DMEM. The cells were washed and then were treated with clostridium perfringens sialidase (0.5 unit ml^{-1}) for 1 h at 37 ℃. The resultant cells were washed and then stained with FITC-SNA for Sia or biotin-conjugated anti-DNP IgG (10 μg ml^{-1}) for 15 min at 4 ℃, stained with PE-labeled strepavidin (10 μg ml^{-1}) for 15 min at 4 ℃ for ^{DNP}Sia-cultured cells. The resultant cells were washed with PBS and then analyzed by flow cytometry.

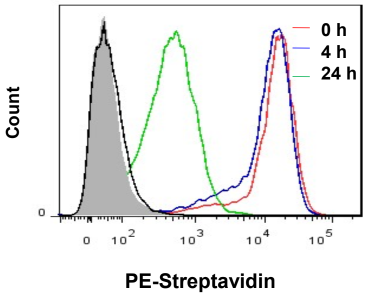

Fig. S2 Temporal retention of ^{DNP}Sia on cell surface. B16F10 cells precultured in DMEM spiked with or without ^{DNP}Sia methyl ester (100 μM) for 24 h were washed and then cultured in fresh DMEM. At inidcated time points, the cells were washed and then stained sequentially with biotin-labeled anti-DNP Ab (10 μg ml^{-1}) for 15 min at 4 ℃ and PE-labeled streptavidin (10 μg ml^{-1}) for 15 min at 4 ℃, and then probed by flow cytometry. The gray shading represented control cells free of ^{DNP}Sia and immunostaining. The black lines showed fluorescence of ^{DNP}Sia free cells that were stained sequentially with biotin-labeled anti-DNP Ab (10 μg ml^{-1}) for 15 min at 4 ℃ and PE-labeled streptavidin (10 μg ml^{-1}) for 15 min at 4 ℃. The green, blue, and red peaks represent immunostaining of DNP-bearing cells that cultured in fresh medium for 0-24 h as indicated.

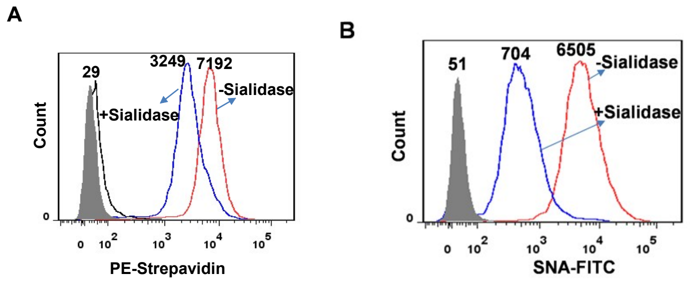

Fig. S3 Rates of enymatic hydrolysis of cell surface ^{DNP}Sia and Sia by sialidase. B16F10 cells displaying cell surface ^{DNP}Sia (A) or natural Sia (B) were treated with sialidase (0.5 unit ml^{-1}) for 1 h at 37 ℃. The resultant cells were washed and then stained with FITC-SNA for native Sia or biotin-conjugated anti-DNP IgG (10 μg ml^{-1}) for 15 min at 4 ℃, stained with PE-labeled strepavidin (10 μg ml^{-1}) for 15 min at 4 ℃ for ^{DNP}Sia. The cells were washed with PBS before flow cytometric analysis. The gray shading represented fluorescence of cells free of immuno-staining. The black lines showed fluorescence of ^{DNP}Sia-free cells that were immuno-stained sequentially.

Western blot analysis of covalent incorporation of ^{DNP}Sia and $^{DNP\text{-}Tz}$Sia into glycoconjugates

B16F10 cells were first cultured for 24 h in DMEM spiked with no sugar, ^{DNP}Sia (0.1, 1 mM) or $^{DNP\text{-}Tz}$Sia methyl ester (0.1, 1 mM). The cells were collected, washed with PBS and then lysed with 1 ml of cell lysis buffer. The mixture were pelleted by centrifugation at 10,000 g for 10 min and the supernatant were diluted with lysis buffer to protein concentration of 1 mg ml^{-1} as determined by BCA protein assay. The diluted samples were respectively added to 2 x SDS-PAGE loading buffer, resolved on 10% SDS-PAGE gels, transferred to nitrocellulose, and blocked with 5% bovine serum albumin in PBST (Dulbecco's Phosphate Buffered Saline with 0.05% Tween-20) for 1 h at 37 ℃. The blocked membrane was incubated with biotin-labeled anti-DNP Ab (1 μg ml^{-1}) in blocking buffer for 2 h at 37 ℃, followed by HRP-conjugated streptavidin (1 μg ml^{-1}) in blocking buffer for 2 h at 37 ℃, washed with PBST (3 x 10 min per wash), and developed using Super ECL Plus Chemiluminescent substrate.

Induction of anti-DNP antibodies in mice with ^{DNP}KLH

C57BL/6 mice were subcutaneously injected at the base of the tail with ^{DNP}KLH (50 μg) in 100 μl TiterMax Gold adjuvant (emulsions prepared according to the manufacturer's instructions). Two weeks later, Mice were further injected with 80% of the primary antigen dose injected on the back of the neck for 14 days after the first injection. The mice were maintained for following experiments.

ELISA analysis for anti-DNP Ab in mice

Blood samples were collected from the immunized mice and the levels of DNP-specific antibodies in the serum were analyzed by enzyme linked immunosorbent assay (ELISA) performed according to manufacturer's recommendation. Briefly, 96-well plates were coated with DNP-conjugated bovine serum albumin (^{DNP}BSA) at doses of 2 μg well^{-1} overnight and then blocked with 5% BSA in PBST for 1 h at 37 ℃. The serum from immunized or control mice were collected 2 weeks after second immunization and diluted using PBS (1:10^4). 100 μL of diluted sera or PBS was added to each well, and the plate was incubated for 1 h at 37 ℃. The plate was washed with PBST and then incubated with a HRP-conjugated goat anti-mouse Ab (100 μL, 1:1000) for 1 h. The plates were washed four times with PBST, followed by addition of 200 μl/well of 3, 3', 5, 5'-tetramethylbenzidine (TMB). The enzymatic reaction was stopped after 5 min in the dark at rt by addition of H_2SO_4 solution (2 M, 50 μL). The optical absorbance at 450 nm was determined using a SpectraMax Microplate Reader (Molecular Devices).

Effects of ^{DNP}Sia on cell proliferation

Dose dependent cytotoxicity: B16F10 cells were cultured in DMEM containing ^{DNP}Sia methyl ester (0, 0.01, 0.1, 1 mM) for 24 h. The cells were washed with PBS and then cultured for another 24 h in fresh DMEM. Cell number and cell proliferation were determined by MTT cell proliferation assay.

Incubation time dependent cytotoxicity. B16F10 cells were cultured in DMEM containing ^{DNP}Sia methyl ester (1 mM) for 0-72 h. At different cell incubation time, a portion of the cells were washed with PBS and then subjected to MTT assay. Cells were treated with MTT reagent for 3 h at 37 ℃. Absorbance at 590 nm was measured. In addition, Cell number was determined by Trypan Blue Assay.

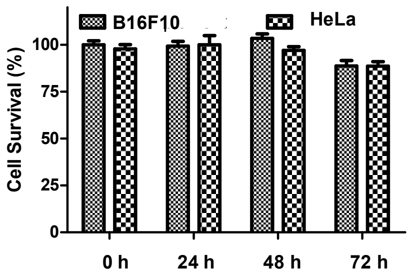

Fig. S4 Cytotoxicity of ^{DNP}Sia. B16F10 cells or HeLa cells were incubated with ^{DNP}Sia methyl ester (1 mM) in DMEM for 0-72 h. At indicated time points cell viability was determined by MTT assay, and then analyzed by MTT assay. The cell viabilities were normalized by control B16F10 cells cultured in the absence of ^{DNP}Sia.

Inhibition of tumor growth in mice mediated by cell surface-anchored ^{DNP}Sia

B16F10 cells were cultured in DMEM spiked without or with ^{DNP}Sia methyl ester (1 mM) for 24 h. The resultant ^{DNP}Sia-displaying B16F10 cells (2.5x10^6) or control ^{DNP}Sia-free B16F10 cells inoculated into both flanks of mice that have been immunized with or without ^{DNP}KLH. The mice were anesthetized 7 days post inoculation, and the tumors were dissected from mice. In parallel experiments, immunized or control mice that have been implanted with ^{DNP}Sia-displaying B16F10 cells (2.5x10^6) or ^{DNP}Sia-free B16F10 cells (control) were maintained. The tumor volumes in living mice were measured over time (3-14 days post-treatment).

Inhibiting B16F10 xenograft formation with intravenously injected ^{DNP}Sia

B16F10 cells (2.5x10^6) were inocualted subcutaneously in immunized C57BL/6 mice for 3 days. The resultant mice were treated with PBS (100 μl) or ^{DNP}Sia (30 mg kg^{-1}) at 3rd, 6th and 9th day after tumor transplantation. The tumor volumes in mice were measured over time (6-14 days after cell implantation).

Inhibition of pulmonary metastasis of B16F10 cells with surface-anchored ^{DNP}Sia

B16F10 cells were cultured in DMEM spiked without or with ^{DNP}Sia methyl ester (1 mM) for 24 h. Eight immunized mice were respectively intraveously injected by tail vein with ^{DNP}Sia-positive B16F10 cells (2.5x10^6), ^{DNP}Sia-negative B16F10 cells (2.5x10^6), or no cells (PBS). The mice were anesthetized at 14th day post injection. The lung and other representative organs were excised and examined for B16F10 metastasis.

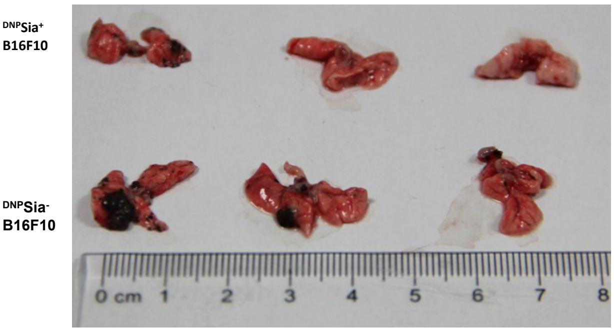

Fig. S5 Images of a group of female mice treated by intravenous injection of ^{DNP}Sia-positive B16F10 cells or ^{DNP}Sia-negative B16F10 cells. Lungs from these mice were excised and then images 14 days post injection.

In vivo distribution of ^{DNP}Sia in tumor-bearing mice

A cohort of C57BL/6 mice was subcutaneously injected with B16F10 cells and then maintained for 5-7 days to allow formation of tumor xenografts. The mice were injected intravenously with ^{DNP}Sia (60 mg kg^{-1}) via tail vein. At 1 h following injection, the mice were anesthetized. The tumors and selected organs were excised, washed with PBS, and then sectioned. The slices were blocked with 5% bovine serum albumin in PBST (Dulbecco's Phosphate Buffered Saline with 0.05% Tween-20) for 30 min at 37 ℃, stained with biotin-labeled DNP-specific Ab (10 μg ml^{-1}) for 30 min at 37 ℃, washed with PBST and then stained with PE-labeled streptavidin (10 μg ml^{-1}) for 30 min and DAPI (1 μM) for 15 min at 37 ℃, and then probed for *ex vivo* PE fluorescence.

Retention of ^{DNP}Sia in tumors in mice

A cohort of C57BL/6 mice bearing subcutaneous B16F10 tumors were injected intravenously with ^{DNP}Sia (60 mg kg^{-1}) via tail vein. At 0-24 h following injection, the mice were anesthetized and the tumor were excised, washed with PBS, and then sectioned. The slices were blocked with 5% bovine serum albumin in PBST for 30 min at 37 ℃, stained with biotin-labeled DNP-specific Ab (10 μg ml^{-1}) for 30 min at 37 ℃, washed with PBST and then stained with PE-labeled streptavidin (10 μg ml^{-1}) for 30 min and DAPI (1 μM) for 15 min at 37 ℃, and then probed for *ex vivo* PE fluorescence.

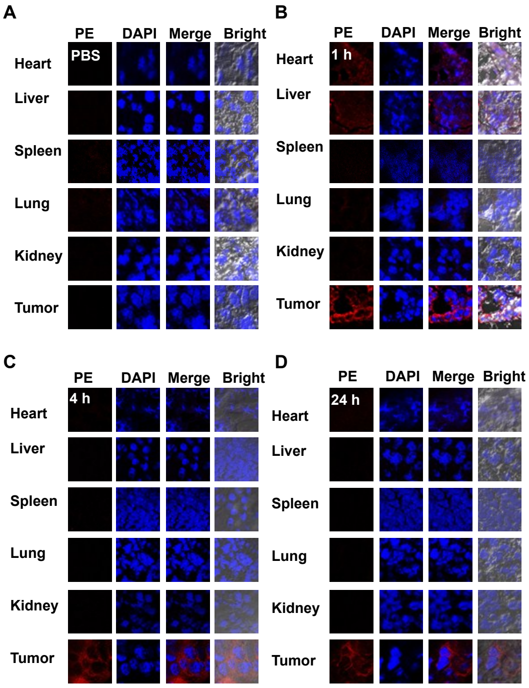

Fig. S6 Temporal retention of ^{DNP}Sia in tumor. C57BL/6 mice bearing subcutaneous B16F10 tumors were injected by tail veil with PBS (A), or ^{DNP}Sia (60 mg kg^{-1}) (B-D). The tumor and organs were excised 1-24 h post-injection, sectioned, and stained with biotin-labeled anti-DNP Ab, PE-labeled streptavidin and DAPI prior to fluorescence analysis.

Cytotoxicity of ^{DNP}Sia *in vivo*

For cell toxicity: B16F10 cells were respectively cultured for 0-72 h in DMEM medium containing ^{DNP}Sia (0-1 mM) as described above. The cell number and cell viability were determined by MTT assay.

For systemic toxicity: Healthy mice were intravenously injected with ^{DNP}Sia (300 mg kg^{-1}) via tail vein. The mice were monitored regularly for adverse physiological effects. At 14 days post-injection, the mice were sacrificed and the tumor and selected organ was harvested, sectioned, and then stained with hematoxylin and eosin, and then recorded for visual images.

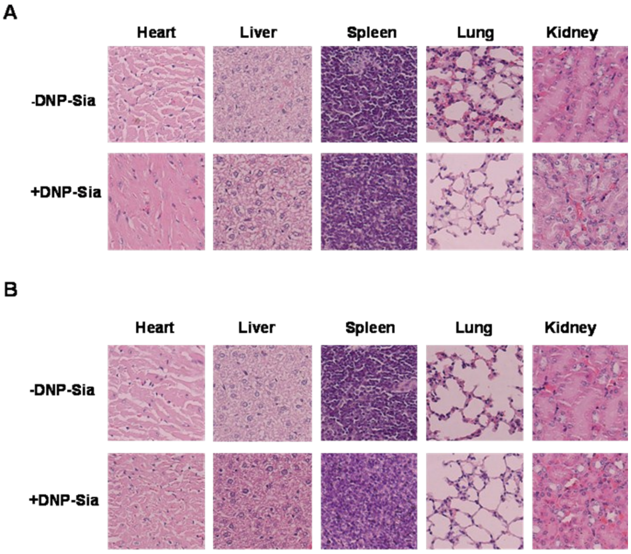

Fig. S7 Histological images of organs from mice intraveously injected by tail vein with ^{DNP}Sia (300 mg kg^{-1}). The mice were sacrificed. The tumor and selected organs were harvested, sectioned, stained with hematoxylin and eosin, and then recorded for visual images.

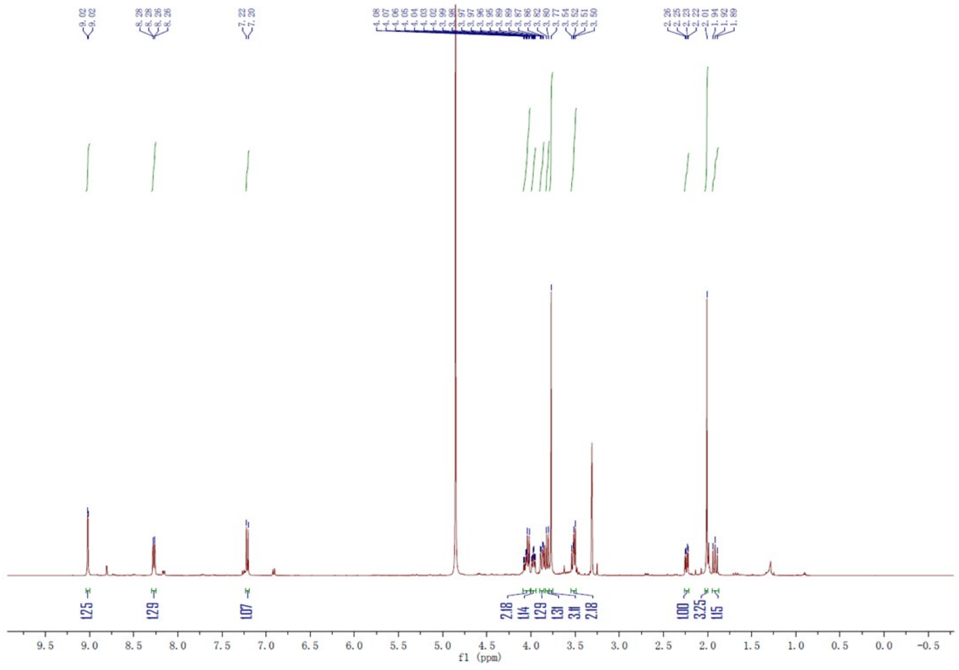

Figure S8. ^{1}H-NMR of ^{DNP}Sia methyl ester.

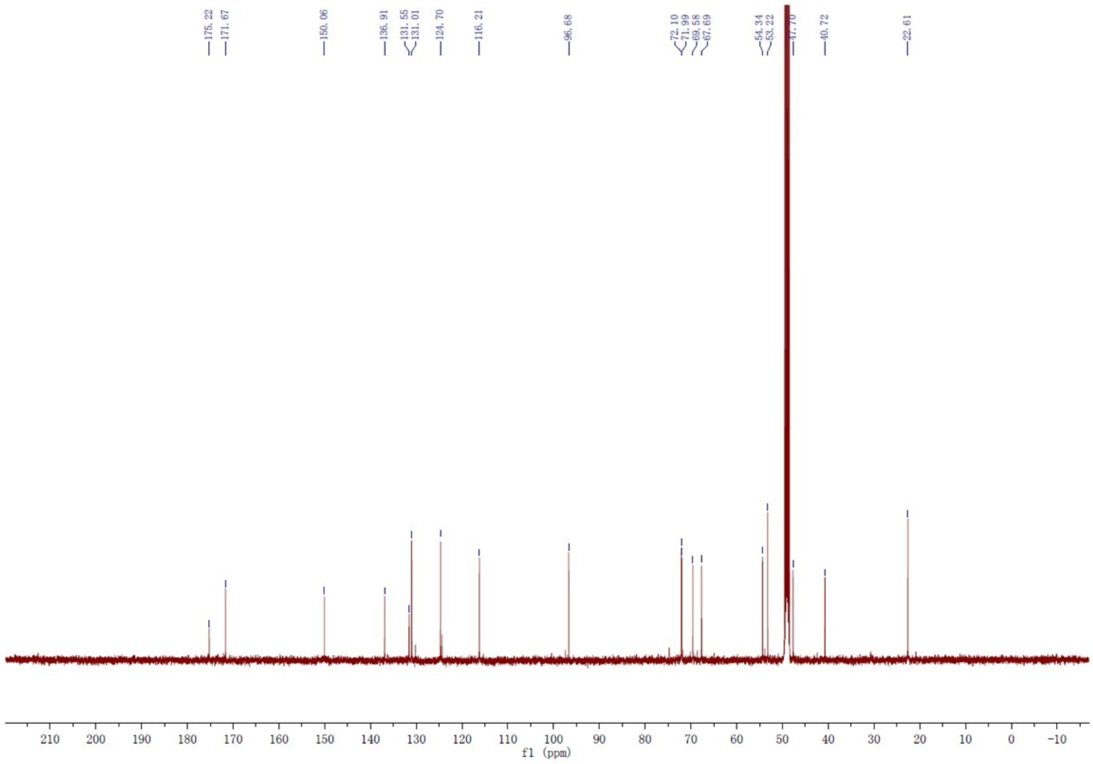

Figure S9. ^{13}C-NMR of ^{DNP}Sia methyl ester.

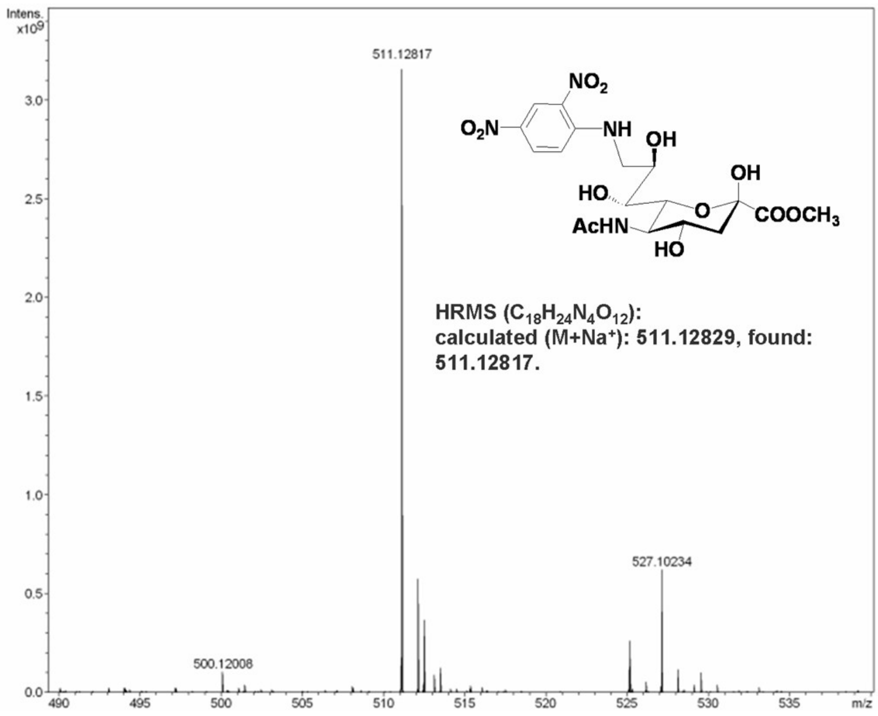

Figure S10. HRMS spectrum of ^{DNP}Sia methyl ester.

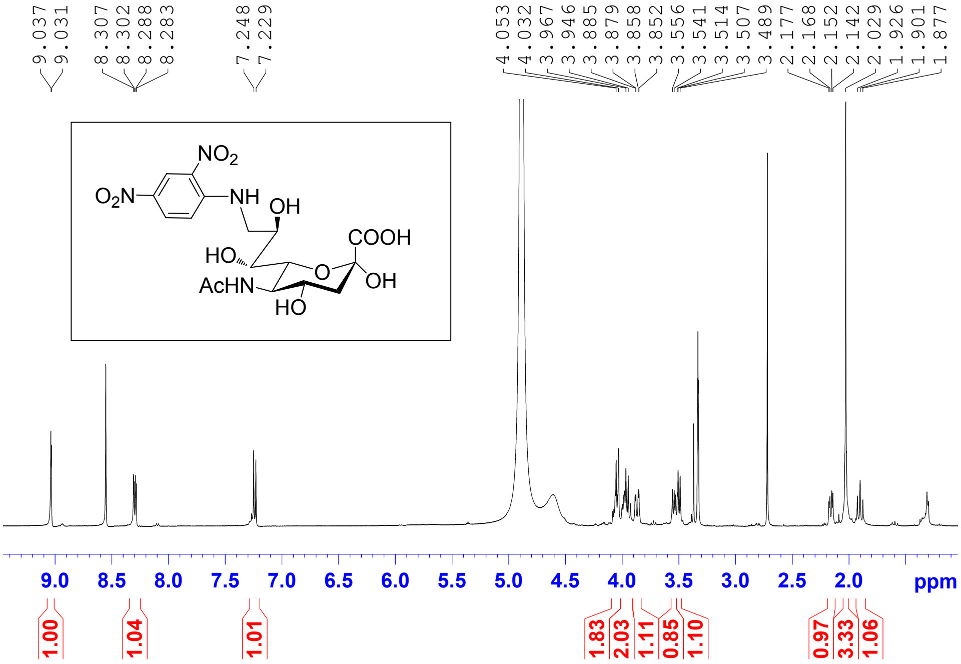

Figure S11. ^{1}H-NMR of ^{DNP}Sia.

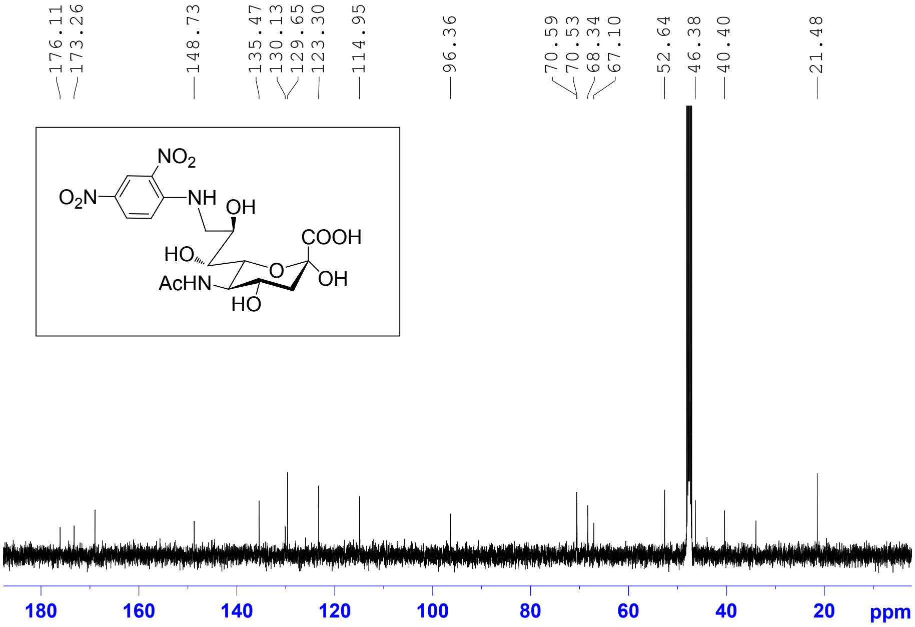

Figure S12. ^{13}C-NMR of ^{DNP}Sia.

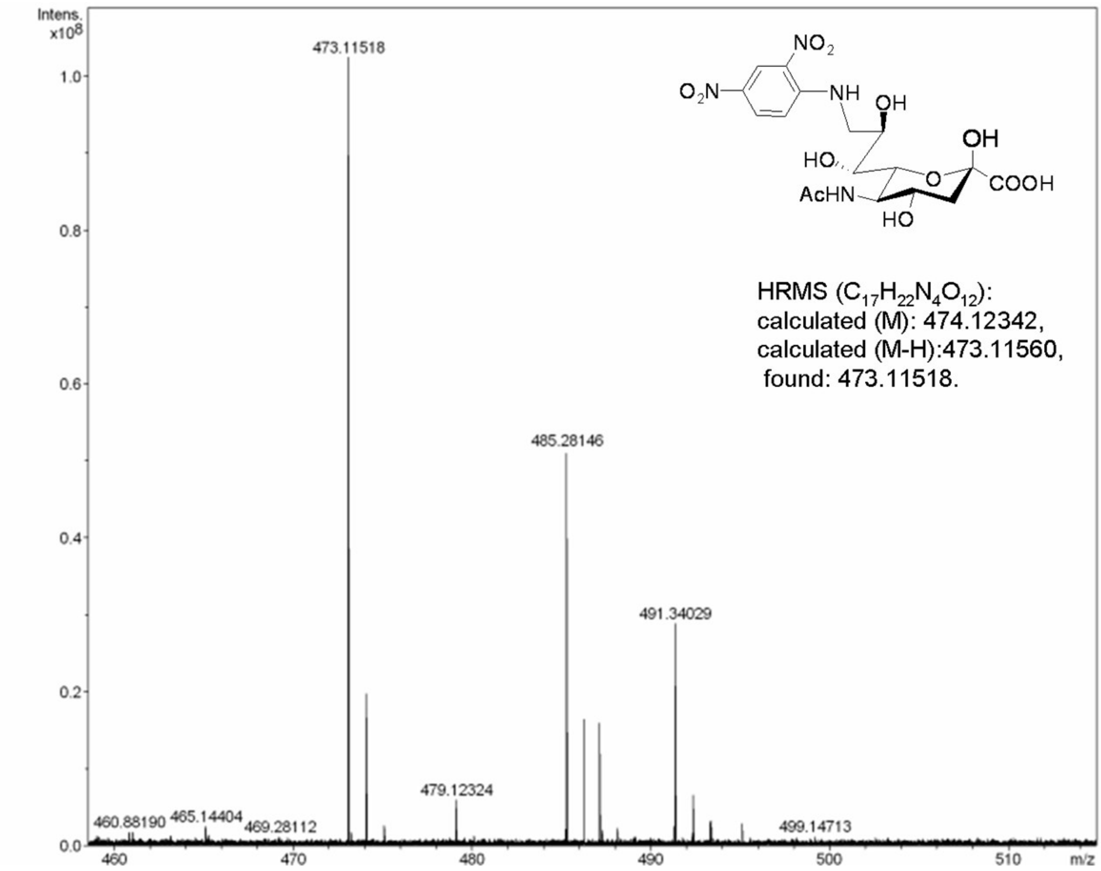

Figure S13. HRMS spectrum of ^{DNP}Sia.

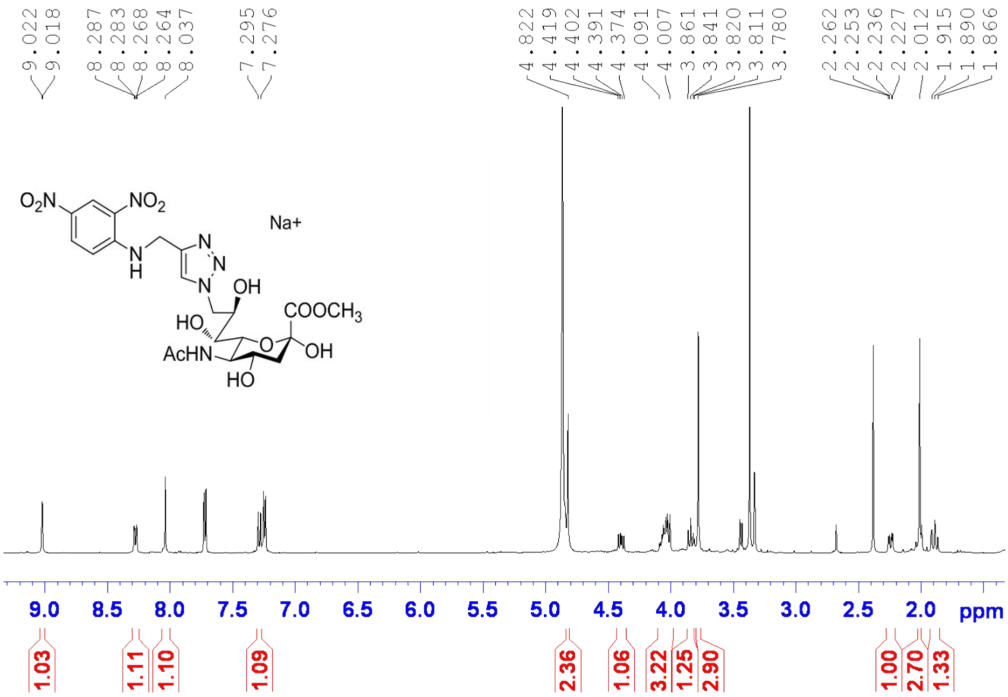

Figure S14. ^{1}H-NMR of $^{DNP\text{-}Tz}$Sia methyl ester.

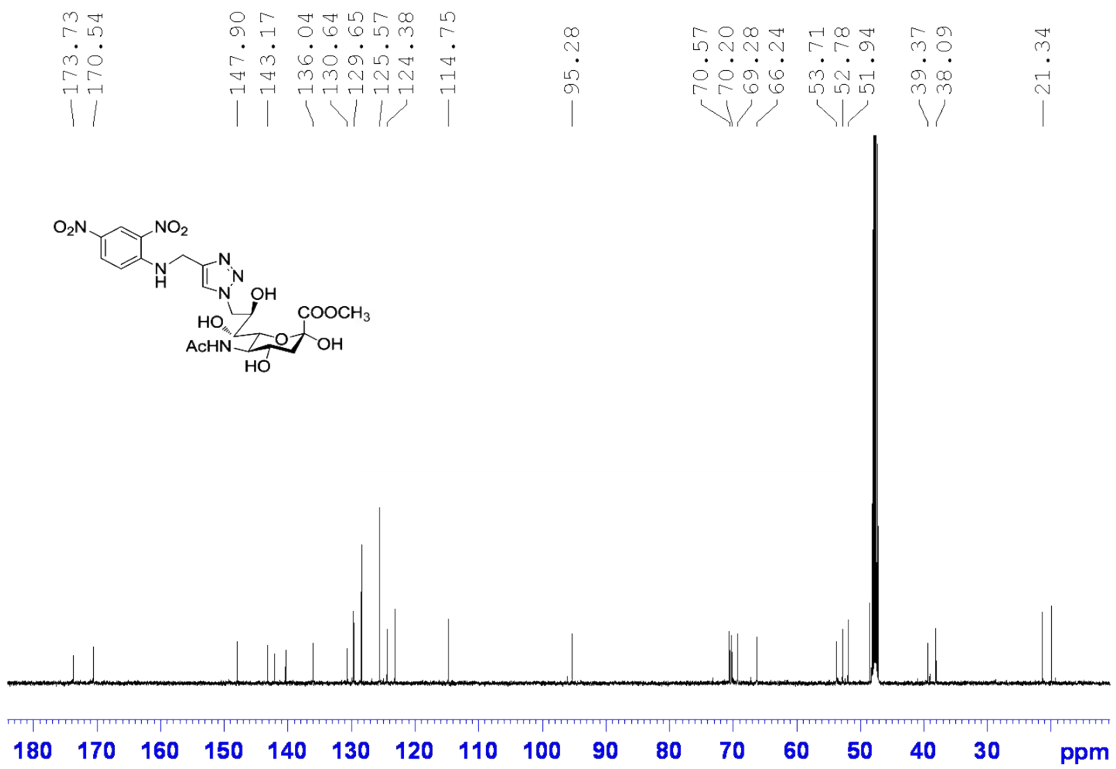

Figure S15. ^{13}C-NMR of $^{DNP\text{-}Tz}$Sia methyl ester.

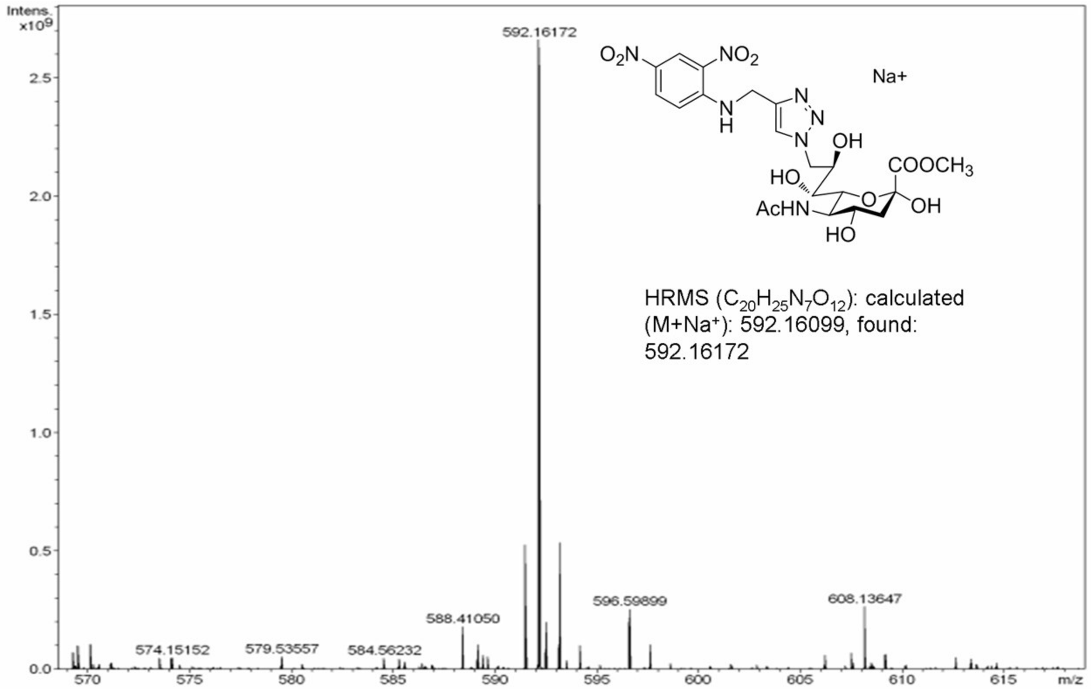

Figure S16. HRMS specteum of $^{DNP\text{-}Tz}$Sia methyl ester.
